# What is Common Becomes Normal; Black-White Variation in the Effects of Adversities on Subsequent Initiation of Tobacco and Marijuana During Transitioning into Adolescence

**DOI:** 10.29245/2578-2959/2024/1.1300

**Published:** 2024-02-08

**Authors:** Shervin Assari, Babak Najand, Payam Sheikhattari

**Affiliations:** 1 Department of Internal Medicine, Charles R Drew University of Medicine and Science, Los Angeles, CA, USA; 2 Department of Urban Public Health, Charles R Drew University of Medicine and Science, Los Angeles, CA, USA; 3 Marginalization-related Diminished Returns Center, Los Angeles, CA, USA; 4 Center for Urban Health Disparities Research and Innovation, Morgan State University, Baltimore, MD, USA; 5 The Prevention Sciences Research Center, School of Community Health and Policy, Morgan State University, Baltimore, MD, USA; 6 Department of Behavioral Health Science, School of Community Health and Policy, Morgan State University, Baltimore, MD, USA

**Keywords:** Race, Socioeconomic position, Stress, Adverse life events

## Abstract

**Background:**

While adversities across domains of finance, race, family, and life may operate as risk factors for initiation of substance use in adolescents, the influence of these factors may vary across racial groups of youth. Unfortunately, the existing knowledge is minimal about racial differences in the types of adversities that may increase the risk of subsequent substance use initiation during the transition into adolescence.

**Aim:**

To compare racial groups for the effects of adversities across domains of finance, race, family, and life on subsequent substance use initiation among pre-adolescents transitioning into adolescence.

**Methods:**

In this longitudinal study, we analyzed data from 6003 non-Latino White and 1562 non-Latino African American 9–10-year-old children transitioning into adolescence. Data came from the Adolescent Brain Cognitive Development (ABCD) study. Participants were followed for up to thirty-six months as they transitioned to adolescence. The independent variables were adversities related to the domains of finance, race, family, and life. The primary outcomes were time to first tobacco or marijuana use. Age, puberty, and gender were confounders. Cox regression models were used for data analysis.

**Results:**

For White youth, tobacco use was under influence of having two parents in the household (HR = .611; 95% CI = .419-.891), parental education (HR = .900; 95% CI = .833-.972), household income (HR = .899; 95% CI = .817-.990), racial stress (HR = 1.569; 95% CI = 1.206–2.039), and life stress (HR =1.098 ; 95% CI = 1.024–1.178) and marijuana use was under influence of neighborhood income (HR = .576; 95% CI = .332-.999) and financial stress (HR =4.273; 95% CI = 1.280–17.422). No adverse condition predicted tobacco or marijuana use of African American youth.

**Conclusion:**

The effects of adversities on substance use depend on race. While various types of adversities tend to increase subsequent initiation of tobacco and marijuana, such factors may be less influential for African American adolescents, who experience more of such adversities. What is common may become normal.

## Introduction

For several reasons, it is essential to explore the intricate dynamics between multiple domains of adversities with subsequent substance use of children transitioning to adolescence across diverse racial groups. First, adversities operate as major social determinants of developmental outcomes in children and adolescents^1^. Children who experience adverse life events will show a higher risk in terms of behavioral and developmental outcomes as they transition to adolescence. Adversities, however, are not uniform and occur across various domains of adolescents’ lives^2,3^. Children and adolescents who experience higher levels of adversity are likely to experience negative brain and neurocognitive outcomes^4^. In addition to anxiety^5^, post-traumatic stress disorder^6^, depression^5^, and suicide^3^, some of the well-established consequences of exposure to adversities are use of various substances^7^ and drugs^8^. Children and adolescents who experience adversities are at increased risk of tobacco^9^, marijuana^10,11^, and alcohol use^12^.

Second, some literature indicates racial group differences in the complex interplay between adverse conditions and health behaviors such as substance use^13^. It is imperative to acknowledge that individual and group responses to various types of adversities are multifaceted and shaped by a broad range of factors such as coping, available resources, preferences, and cultures, as well as the presence of other risk and resilience factors such as social support, which are all different across diverse racial groups^14^. Ongoing research in this field continues to evolve, providing valuable insights into the complex interplay between stress, and substance use within diverse populations^15^.

Third, it is possible to expect racial differences in the response to adversities^16,17^. In other words, it is essential to test whether habituation^18^ or sensitization^19^ results in group variation in response to adverse conditions. On one side, habituation suggests that prolonged exposure to stressors may lead individuals to become desensitized or less responsive to the effects of stress over time. In the context of racial groups, such as African Americans and other minority communities, habituation implies a potential attenuation of the effects of stress, resulting in a lower impact^18^. Conversely, sensitization proposes that the effects of adversity may be more pronounced or stronger for certain disadvantaged groups. According to the cumulative disadvantage hypothesis, first exposure may increase sensitivity to the same exposure in the future^19^. This implies that due to repeated exposure to adversities, racial minority groups could experience an intensified impact on substance use^20^. According to this hypothesis, disadvantaged racial groups would be more vulnerable to the negative consequences of adversity, as proposed by cumulative disadvantage^20^, double disadvantage^21^, life course epidemiology^22^, and weathering^23^ hypotheses.

Fourth, as there are very few studies on race differences in the effects of adversities on tobacco and marijuana use, we tested the intricate relationship between stress, race, and substance use across racial groups, whose lives differ by a myriad of factors. Understanding the nuanced differences in the effects of various adversities on the use of each substance in adolescents is essential because such nuanced information may be important for the development of effective interventions and support systems^24^. Recognition of these dynamics holds particular significance for public health initiatives and policies^25,26^. Tailoring interventions to account for the unique experiences of different racial groups becomes essential in addressing health disparities related to the use of various substances^27^. Implication of such knowledge will be increased cultural sensitivity in substance use prevention^27–29^.

Fifth, racial minority status^30^ and low socioeconomic position (SEP)^31^ are two overlapping constructs^32^. Racial minority and low-SEP families report high levels of exposure to adverse life experiences during childhood as well as the transition to adolescence^32^. As exposure to adverse life experiences connects race and SEP to health and development^33^, higher exposure to adverse life experiences during childhood and transition to adolescence may be one of the main underlying mechanisms that explain racial- and SEP-related disparities in health and development^32^. There is some evidence suggesting that adverse life experiences may explain why SEP impacts population and individual health^34^.

Sixth, due to the legacy of slavery^35^ and contemporary racism^36^, social determinants are racialized^37–41^. This means access to social determinants and effects of such resources on health and behaviors differ by race. Not only exposures to adversities are higher in minoritized populations^42^, minorities show diminished effects of various economic resources and adversities on behavioral and developmental outcomes^43,44^, such as tobacco^45^, marijuana^46^, and alcohol use^47,48^. African American youth and adults show diminished protective effects of certain family economic status on tangible health and developmental outcomes^49,50^, a pattern showed for various financial and economic indicators such as family income^48^, and parental SEP^51–53^. However, most of the weaker effects of economic status on behavioral outcomes are cross-sectional^43,44,50,54,55^.

Thus, there is a need for longitudinal comparisons of diverse racial groups on the effects of exposure to adverse financial conditions on tobacco use. To give a few examples, in the Adolescent Brain Cognitive Development (ABCD) study data that included 9–10-year-old children, SEP effects on exposure to adverse life experiences were weaker for African Americans than non-Latino Whites. That study, however, it was a cross-sectional study without follow-up of participants over time, and only compared the role of parental education and family income and did not include the role of family structure^56^. A cross-sectional study using data from the Fragile Families and Child Wellbeing Study (FFCWS) showed a weaker inverse link between SEP and spanking in African Americans than in non-Latino White children^57^. Across all SEP levels, African American parents reported higher levels of occupational stress, while non-Latino White parents reported the least number of occupational stress^58^. In a cross-sectional survey, at each level of SEP, African American families remained at higher risk of poverty than non-Latino Whites^53,59^. However, past research has been mainly cross-sectional, and there is need for longitudinal research.

Breaking down these concepts, it becomes evident that while adversities yield considerable influence over the use of tobacco and marijuana, these effects may differ across diverse social groups who may have different coping mechanisms when adversities are faced. Cultural differences, socioeconomic status, and historical contexts contribute to variations in how stress influences substance use across diverse racial communities.

Previous cross-sectional studies have recently shown stronger effects of SEP on reducing exposure to adverse life experiences among non-Latino Whites than racial minority children, youth, and adults^60–63^. However, we are unaware of any past research on longitudinal associations between race, SEP, adversities, and behaviors.

### Aims

Our aim was to compare racial groups for the effects of exposure to adverse life experiences on future tobacco and marijuana use of children during their transition to adolescence. We expected weaker effects of adverse life experiences during the transition to adolescence for racial minorities than non-Latino White children. Our expected differential sensitivity of non-Latino White and African American adolescents to adverse life experiences during the transition to adolescence is based on observations on racial differences in the effects of SEP on youth who experience depression, anxiety, and substance use regardless of their resources and assets and SEP^43,44^. The unique contribution of this paper is the longitudinal design and expansion of the comparison of SEP indicators to advertise the inclusion of multiple types of adversities.

## Methods

We conducted a secondary analysis of the Adolescent Brain Cognitive Development (ABCD) study data. ABCD is a national longitudinal investigation of a racially and economically diverse sample of pre-adolescent children transitioning to adolescence^64^. More information about ABCD study methodology is available elsewhere^64^. Some of the advantages of the ABCD data include longitudinal, national, large, and diverse samples in terms of race, SEP, and geography. *T*he ABCD recruitment was mainly from schools^65^. The analytical sample was composed of 6003 non-Latino White and 1562 non-Latino African American pre-adolescents. The ABCD project ethics was approved by the Institutional Review Board (IRB) of the University of California, San Diego (UCSD). All adolescents who participated in the ABCD project provided assent. All parents who participated in the ABCD project signed informed consent^66^.

The study variables included race, demographic and socioeconomic factors, adversities, and substance sue.

### Socioeconomic Status

#### Number of Parents Present in the Household.

Parents reported the number of parents in the household. This variable was operationalized as a categorical variable with 0 for one and 1 for two-parent households.

#### Family Income.

Family income was a 1–10 interval measure where a higher score indicated higher income. The total combined family income in the past 12 months was asked. Responses were 1 = less than $5000; 2 = $5000+; 3 = $12,000+; 4 = $16,000+; 5 = $25,000+; 6 = $35,000+; 7 = $50,000+; 8 = $75,000+; 9 = $100,000+; and 10 = $200,000+. This categorization was made by the ABCD study. This variable was coded as a continuous measure.

#### Parental Educational Attainment.

Participants were asked, “What is the highest grade or level of school you have completed or the highest degree you have received?” Responses were 0 = Never attended/Kindergarten only; 1 = 1st grade; 2 = 2nd grade; 3 = 3rd grade; 4 = 4th grade 4; 5 = 5th grade; 6 = 6th grade 6; 7 = 7th grade 7; 8 = 8th grade; 9 = 9th grade; 10 = 10th grade 10; 11 = 11th grade; 12 = 12th grade; 13 = high school graduate; 14 = GED or equivalent diploma; 15 = some college; 16 = associate degree: occupational; 17 = associate degree: academic program; 18 = bachelor’s degree; 19 = master’s degree; 20 = professional school degree; and 21 = doctoral degree. This variable was an interval measure with a range between 1 and 21, with a higher score indicating higher parental educational attainment.

#### Neighborhood Income.

Using zip code data, ABCD has collected median family income in the zip code. We used this variable after dividing it by 5000 to have more understandable beta coefficients.

#### Race (Moderator).

Parents reported race. This variable was a categorical measure with the following categories: non-Latino African American and non-Latino White (reference category).

### Predictors

#### Life Stress.

Adverse life experiences during the transition to adolescence were measured at baseline during an interview using the Life events history, which is a validated instrument to measure traumatic events and adversities. Life history is a semi-structured interview with items such as 1) Someone in family died?, 2) Family member was seriously injured?, 3) Saw crime or accident?, 4) Lost a close friend? The response for each item was 0 (no) or 1 (yes). If an even had experienced, then follow up questions were asked as the following: If the event had happened during the last year, if the even was negative or positive, and to what degree the individual’s life was affected. Given all these items, we calculated the total adverse life experiences during the transition to adolescence every two years. So, we calculated the events that happened in the last year, were found to be negative, and had an impact on the individual. By counting the responses that indicated the negative effect of the adverse life experience on the child (0 = Not at All; 1 = A Little; 2 = Some; 3 = A lot), we had a variable as a continuous measure with higher scores indicating higher exposure to adverse life experience impact during the transition to adolescence^67^. Appendix 1 shows the calculation of the score. As this formula shows, we calculated the adverse life events’ sum score (total number).

#### Financial Stress.

Subjective family SES in this study was financial difficulties measured by the following seven items: “In the past 12 months, has there been a time when you and your immediate family experienced any of the following:” (1) “Needed food but could not afford to buy it or could not afford to go out to get it?”, (2) “Were you without telephone service because you could not afford it?” (3) “Did you not pay the full amount of the rent or mortgage because you could not afford it?”, (4) “Were you evicted from your home for not paying the rent or mortgage?”, (5) “Had services turned off by the gas or electric company, or did the oil company not deliver oil because payments were not made?”, (6) “Had someone who needed to see a doctor or go to the hospital but did not go because you could not afford it?”, and (7) “Had someone who needed a dentist but could not go because you could not afford it?” Responses to each item were either 0 or 1. We calculated a mean score with a potential range between 0 and 1—a higher score indicating higher subjective family SES. Our variable was a continuous measure^68,69^.

#### Racial Stress.

Perceived discrimination was measured using seven items, at the end of one year follow up. One example item was “How often do the following people treat you unfairly or negatively because of your ethnic background?” Items ranged from 1 to 5, with 1 referring to = almost never and 5 referring to very often. Participants were allowed to say they don’t know or refuse to answer. The total score was an average of the items, ranging from 1 to 5, with a higher score indicating more discrimination^70^.

#### Family Stress.

Family conflict in this study was measured using the Family Environment Scale with nine items^71^. To complete this measure, adolescents report negative aspects of their relations with their parents and family. Items of this questionnaire measure the extent of fighting, anger, criticism, competitiveness, yelling, and/or loss of temper within the family^71^. The measure provides a continuous score with a higher score indicating higher conflict in the family. Cronbach’s alpha of the family conflict measure was 0.681 overall. We calculated a mean score where high score was indicative of conflicting relation with the family^72^.

### Outcome

#### Substance Use.

Annual assessments of tobacco and marijuana use involved the iSay Sipping Inventory^73^ for recent or first experimentation with nicotine products^74^. Follow-up questions on circumstances surrounding first use were administered at one time-point, however, such information was not included in our analysis. At Baseline (Y0), adolescents reported lifetime use of substances with a web-based Timeline Follow-Back (TLFB)^75^ interview for substances used in the past six months (only for baseline evaluation at time 0) or since the last study session (for measures at months six, twelve, eighteen, twenty four, and thirty months). The analysis covered various substances, and Mid-Year phone follow-ups contributed to a comprehensive past-year substance use for each yearly follow-up. For the current analyses, substance use variables were defined as follows: Substance use at the level of experimentation included low-level tobacco (puffing) or marijuana use (e.g., puffing). Substance use initiation, which was the outcome considered here, was reporting >1 puff of nicotine or marijuana. We created three separate variables that reflected new onset of tobacco and marijuana use that were captured six months or later after baseline.

### Data Analysis

For the purpose of this analysis, we used the SPSS statistical package. For univariate analysis, we reported the mean and standard deviation [SD] of our continuous measures overall and by race. We also reported frequency/percentage overall and by race. Then, we reported Pearson correlation tests by race. For multivariable analysis, Cox regression models were used. These models calculated the time to initiation of each substance as the event. Cox regression models were performed in specific racial groups. We tested Cox proportional hazard assumption. No variable required transformation. The outcomes were time to tobacco and marijuana initiation during the transition to adolescence. Predictors were various types of adversities. The moderator was race. Adverse life experiences at baseline (predictors) were continuous measures. Confounders were age, gender, and family SEP (parental education, number of parents). Before we tested the models, we ruled out collinearity between our measures. There was no collinearity between our study variables (all correlations weaker than .6). Hazard Ratio (HR), 95% confidence interval (CI), and p-value were reported.

## Results

Our analysis included 6003 non-Latino White and 1562 non-Latino African American 9–10 years old children being followed over time for up to 36 months. African American and non-Latino White adolescents differed in gender, family education, income, and family composition, as well as adverse experiences during the transition to adolescence. African American adolescents had lower SEP and higher adversities compared to non-Latino White adolescents. Initiation of tobacco did not vary by race; however, African American adolescents were more likely to initiate marijuana over the follow up. Table 1 shows descriptive data by race.

Table 2 shows bivariate correlations by race. For Whites, tobacco use initiation was associated with all study variables with the exception of number of parents in the household. The only correlation of tobacco use was with marijuana use in African American adolescents. The results show that most bivariate tests are statistically significant, however, the size of the effect is very small for most coefficients (less than 0.1). Exceptions are stronger associations within SES indicators, adversities, and tobacco and marijuana use. These small effect sizes suggest that the strength of the relationship across domains of variables is very weak for marijuana and nicotine use.

### Tobacco initiation

As shown by Table 3, having two parents in the household (HR = .611; 95% CI = .419-.891), parental education (HR = .900; 95% CI = .833-.972), household income (HR = .899; 95% CI = .817-.990), racial stress (HR = 1.569; 95% CI = 1.206–2.039), and life stress (HR =1.098 ; 95% CI = 1.024–1.178) were predictive of future initiation of tobacco for White adolescents. No factor was predictive of future tobacco use for African American adolescents (p > 0.05).

### Marijuana initiation

As shown by Table 4, neighborhood income (HR = .576; 95% CI = .332-.999) and financial stress (HR =4.273; 95% CI = 1.280–17.422) were predictive of future initiation of marijuana for White adolescents. No factor was predictive of future marijuana use for African American adolescents (p > 0.05).

## Discussion

We could find association between various types of adversities and future tobacco and marijuana initiation of non-Latino White but not African American adolescents. Our findings were supportive of habituation rather than sensitization of African American youth against adversities.

We did not find any specific type of adversity that shows consistent effect on all substances even for White youth. For tobacco use, having two parents in the household (HR = .611; 95% CI = .419-.891), parental education (HR = .900; 95% CI = .833-.972), household income (HR = .899; 95% CI = .817-.990), racial stress (HR = 1.569; 95% CI = 1.206–2.039), and life stress (HR =1.098 ; 95% CI = 1.024–1.178) and for marijuana, neighborhood income (HR = .576; 95% CI = .332-.999) and financial stress (HR =4.273; 95% CI = 1.280–17.422) were predictive. As such, the effects of adversities on substance use depend on the type of adversity as well as type of substance in addition to race.

We found adversities to increase substance use of White but not African American youth. This can be explained by the idea that “what is common becomes normal”^76^. African American communities and individuals’ responses to stress may have changed because stress is common^77^. For non-Latino Whites, who experience stress and adversity less frequently, these challenges may be perceived as more salient and impactful. In contrast, African American adolescents may encounter a higher prevalence of stressors in their environment, leading to the normalization of these challenges. This normalization, in turn, may contribute to the development of resilience as a coping mechanism^77^. Resilience within the context of African American adolescents involves the cultivation of adaptive skills and coping strategies in response to the chronic exposure to stress. The constant presence of stressors, whether related to socioeconomic disparities, racial discrimination, or other systemic challenges, necessitates the development of resilience as a survival mechanism^78^. In this regard, African American adolescents may develop a heightened ability to navigate and cope with stress, which could mitigate the association between adversity and substance use^79^. Moreover, the concept of resilience in the face of adversity is deeply rooted in the socio-cultural context of African American communities^80^. Historical and societal challenges due to social stratification, segregation, and systemic racism have shaped a collective resilience that is passed down through generations, fostering a sense of strength and determination. This cultural resilience can act as a protective factor against the negative effects of stressors, including the potential for substance use. To better understand the complex relationship between stress, resilience, and substance use in African American adolescents, it is crucial to consider the multifaceted nature of their experiences. Recognizing the adaptive mechanisms developed within this community provides insights into how cultural factors can influence behavior and contribute to the divergence in the impact of adversity on substance use compared to other racial and ethnic groups^81,82^.

Differential associations between SEP resources, adversities, and behaviors by race are known across age groups^43,44^. The literature has frequently documented differential associations between SEP indicators such as family income and family structure and a wide range of outcomes such as substance use^48,83–85^. This paper suggests that exposure to the same level of adverse life experiences during childhood and transition to adolescence may have larger effects on the substance use of African American than White adolescents. Past work has shown differential effects of SEP on anxiety, depression, suicide, and health problems in racial minority and majority children^48,83–85^ and adults^62,63,86–88^. This result is important because adverse life events contribute to the development of poor behavioral, physical, and mental health outcomes^7,89,90^.

One potential explanation for our finding is segregation. African American families may have a higher tendency to remain in poor neighborhoods compared to non-Latino White individuals. African American families are more likely to have children attending poor schools across SEP levels^91,92^. As a result of residing in high-risk environments, African American adolescents may remain continuously exposed to various types of stressors across SEP levels, compared to non-Latino Whites. We already know that adolescents are exposed to a wide range of chronic stressors in high-risk, low-resource social contexts such as those in a large proportion of African American families^93,94^.

While racialization of social determinants on health and behaviors is well described, the societal and contextual processes that explain such differential effects are still unknown. Researchers have attributed the differential effects of social determinants of health to structural and institutional racism^43,95^. Childhood poverty may reduce the protective effect of SEP when the individual is an adult^96^. Prejudice and discrimination also interfere with the effects of SEP and stress for minority families^63,87,88^. Multilevel economic and societal mechanisms may explain such differential effects of social determinants by race across generations^43,95^.

### Implications

Based on our findings, the following policies and directions can be suggested to reduce exposure and susceptibility to adverse life events in the lives of African American adolescents. We need to equalize the social context of population groups: Segregation and Racism are key factors that may alter the effects of stress and SEP for racial minorities. Therefore, it is important to work towards reducing racism and discrimination against racial minority groups, particularly African Americans. Policy efforts should promote equity and inclusion in all aspects of life, particularly employment. The second is strengthening social support available for racial minority adolescents across SEP lines. Social support is an essential factor in reducing stress and promoting mental health. Policies and programs should focus on increasing access to social support networks for racial minority adolescents. This can be achieved through programs that promote community involvement, peer mentoring, and family engagement. The third is to provide resources for coping with stress. Policies and programs may provide resources and support for dealing with stress in African American communities, neighborhoods, and schools. This can include mental health services, stress management programs, and educational resources that improve adolescents’ coping strategies. The fourth is to improve access to counseling at schools. Policies should focus on improving access to free psychological services in urban areas with a high concentration of African American families. Last but not least, we should educate parents and providers regarding the high prevalence of stress in the lives of African American and non-Latino White adolescents.

### Limitations

This study had a few methodological limitations. We only focused on African American and non-Latino whites, not other racial and ethnic groups or other groups with marginalizing identities. Sexual orientation, nativity, citizenship, and gender identity are other marginalizing identities and may alter the effects of social determinants and adversities^83,97–99^. In addition, we did not have access to other SEP indicators, such as wealth. The study also did not investigate any intersectionality between race and gender in this population. In addition, we did not perform multiple comparison corrections. The difference in numbers between white and African American children was a potential limitation. Culturally relevant factors were not included in this study. Religion involvement, parenting, social support, and other traditions could have played a role. Finally, our analysis failed to point to any specific factors, such as segregation or neighborhood conditions, as possible bases for these differential effects of adversities by race.

### Future Research

Future research may test if neighborhood, school, family, or segregation explain differential sensitivity to adverse life experiences. By including contextual data from neighborhoods, schools, friends, and families, we would better understand what factors cause differential effects of social determinants by race. Research may also investigate the interplay of cultural factors, resilience, and the normalization of stress within different racial and ethnic groups^100^. The discrepancy in the impact of adversity on substance use between non-Latino Whites and African Americans can be better understood through the lens of cultural perspectives and coping mechanisms. We emphasize the importance of adopting a culturally sensitive approach in research and interventions aimed at addressing substance use and mental health issues among diverse populations^101^.

## Conclusion

African American and non-Latino White adolescents exhibit varying levels of sensitivity to diverse adverse life experiences during their transition to adolescence, particularly concerning substance use. The disparate impact of exposure to different types of adverse life experiences may play a role in contributing to racial disparities in substance use among adolescents in the United States. While the ideal scenario would involve reducing all forms of adverse life events for all adolescent groups, our findings highlight specific adversities that could be targeted for prevention efforts, particularly in relation to tobacco and marijuana use among diverse racial adolescents. Adopting a nuanced approach that considers these specific factors may enhance the effectiveness of prevention strategies aimed at curbing substance use among diverse groups of youth.

## Figures and Tables

**Table 1:** Descriptive Data by race (n=7565)

	All			White		African American
	n	%	n	%	n	%
Race						
White	6003	79.4				
African American	1562	20.6				
Gender*						
Girl	3580	47.3	2797	46.6	783	50.1
Boy	3973	52.5	3196	53.2	777	49.7
Missing	12	.2	10	.2	2	.1
Parents in the household*						
One	1822	24.1	886	14.8	936	59.9
Two	5689	75.2	5104	85.0	585	37.5
Missing	54	.7	13	.2	41	2.6
Tobacco Use Initiation						
No	7301	96.5	5792	96.5	1509	96.6
Yes	263	3.5	210	3.5	53	3.4
Missing	1	.0	1	.0		
Marijuana Use Initiation*						
No	7336	97.0	5847	97.4	1489	95.3
Yes	129	1.7	93	1.5	36	2.3
Missing	100	1.3	63	1.0	37	2.4
			**Mean**	**SD**	**Mean**	**SD**
Age	9.492	0.507	9.492	0.505	9.494	0.511
Parental Education*	17.083	2.312	17.564	1.978	15.230	2.561
Household Income*	7.600	2.251	8.187	1.676	5.077	2.633
Neighborhood Income*	0.762	0.426	0.866	0.341	0.365	0.481
Racial Stress*	1.180	0.415	1.130	0.334	1.388	0.609
Financial Stress*	0.058	0.149	0.036	0.117	0.145	0.213
Life Stress*	0.516	1.075	0.472	1.102	0.685	0.948
Family Stress*	0.734	1.019	0.692	0.986	0.897	1.122

**Note.** Source: Adolescent Brain Cognitive Development (ABCD) study. Participants = 6003 non-Latino White and 1562 non-Latino African American 9–10-year-old children.

* p<0.05 for comparison of non-Latino White and African American adolescents

**Table 2:** Bivariate correlations by race

	2	3	4	5	6	7	8	9	10	11	12
**White**											
1 Age											
2 Gender (Boy)	0.019										
3 Two Parents	0.003	−0.001		--							
4 Parental Education	−0.011	−0.025	.166**								
5 Household Income	.027*	−0.008	.419**	.488**							
6 Neighborhood Income	0.022	.031*	.061**	.136**	.204**						
7 Racial Stress	−0.024	.058**	−.057**	−.136**	−.167**	−.048**					
8 Financial Stress	−0.011	0.013	−.192**	−.251**	−.407**	−.114**	.144**				
9 Life Stress	−0.002	0	−.114**	−.095**	−.160**	−.075**	.075**	.151**			
10 Family Stress	0.015	.049**	−.047**	−.054**	−.074**	−.032*	.054**	.139**	.076**		
11 Marijuana Use	.043**	.028*	−0.024	−.048**	−.048**	−.046**	.041**	.054**	.037**	.056**	
12 Nicotine Use	.055**	0.004	−.072**	−.098**	−.109**	−.029*	.079**	.066**	.065**	.121**	.218**
**African American**											
1 Age											
2 Gender (Boy)	0.026										
3 Two Parents	0.017	0.016									
4 Parental Education	0.044	0.003	.219**								
5 Household Income	.067*	−0.021	.428**	.588**							
6 Neighborhood Income	.053*	−0.008	.179**	.308**	.368**						
7 Racial Stress	−0.048	.119**	0.025	−0.046	−.076**	−.059*					
8 Financial Stress	0.011	0.039	−.128**	−.156**	−.265**	−.136**	0.052				
9 Life Stress	0.026	0.005	−.067**	0.017	−0.03	−0.018	−0.033	.181**			
10 Family Stress	−0.031	.068**	−.092**	−.128**	−.164**	−.091**	.065*	.256**	.145**		
11 Marijuana Use	0.043	0.008	−.068**	−0.033	−.082**	−0.046	.068*	0.042	−0.002	.116**	
12 Nicotine Use	0.034	0.04	−0.002	−0.006	−0.021	−0.01	0.017	−0.007	−0.038	.174**	.253**

**Note.** Source: Adolescent Brain Cognitive Development (ABCD) study. Participants = 6003 non-Latino White and 1562 non-Latino African American 9–10-year-old children.

* p<0.05

** p<0.001 using Pearson Correlation test

**Table 3:** Cox regression of time to tobacco use initiation by race

	B	SE	HR	95% CI for HR		p
**White**						
Age	.507	.149	1.661	1.239	2.226	.001
Gender (Boy)	−.064	.150	.938	.699	1.260	.671
Two Parents	−.492	.192	.611	.419	.891	.010
Parental Education	−.105	.039	.900	.833	.972	.007
Household Income	−.106	.049	.899	.817	.990	.030
Neighborhood Income	−.077	.205	.926	.620	1.383	.707
Racial Stress	.450	.134	1.569	1.206	2.039	.001
Financial Stress	.387	.491	1.472	.562	3.855	.431
Life Stress	.094	.036	1.098	1.024	1.178	.009
Family Stress	.108	.068	1.115	.975	1.274	.112
**African American**						
Age	.356	.321	1.428	.761	2.681	.268
Gender (Boy)	.511	.339	1.668	.859	3.239	.131
Two Parents	.026	.385	1.027	.483	2.184	.946
Parental Education	−.032	.079	.968	.829	1.131	.685
Household Income	−.030	.087	.971	.818	1.152	.733
Neighborhood Income	−.112	.370	.894	.433	1.848	.763
Racial Stress	−.009	.259	.991	.596	1.647	.972
Financial Stress	.266	.772	1.304	.287	5.923	.731
Life Stress	−.376	.221	.687	.445	1.059	.089
Family Stress	.057	.143	1.059	.800	1.403	.689

**Note.** Source: Adolescent Brain Cognitive Development (ABCD) study. HR = Hazard Ratio; Participants = 6003 non-Latino White and 1562 non-Latino African American 9–10-year-old children.

**Table 4:** Cox regression of time to marijuana use initiation by race

	B	SE	HR	95% CI for HR		p
**White**						
Age	.599	.229	1.819	1.163	2.847	.009
Gender (Boy)	.442	.236	1.556	.980	2.472	.061
Two Parents	.065	.334	1.067	.555	2.053	.846
Parental Education	−.078	.062	.925	.819	1.045	.212
Household Income	−.004	.082	.996	.849	1.168	.958
Neighborhood Income	−.551	.281	.576	.332	.999	.050
Racial Stress	.307	.236	1.359	.856	2.158	.193
Financial Stress	1.552	.666	4.723	1.280	17.422	.020
Life Stress	.089	.055	1.093	.981	1.219	.107
Family Stress	.050	.106	1.052	.854	1.295	.635
**African American**						
Age	.938	.406	2.555	1.153	5.662	.021
Gender (Boy)	.137	.389	1.147	.535	2.457	.724
Two Parents	−.481	.506	.618	.229	1.669	.343
Parental Education	−.039	.091	.962	.804	1.150	.668
Household Income	−.070	.103	.932	.762	1.141	.496
Neighborhood Income	−.473	.493	.623	.237	1.639	.338
Racial Stress	.390	.228	1.478	.945	2.311	.087
Financial Stress	.726	.807	2.068	.425	10.056	.368
Life Stress	−.163	.220	.849	.552	1.308	.458
Family Stress	.145	.157	1.157	.850	1.574	.354

**Note.** Source: Adolescent Brain Cognitive Development (ABCD) study. HR = Hazard Ratio; Participants = 6003 non-Latino White and 1562 non-Latino African American 9–10-year-old children.

## Data Availability

Data used in the preparation of this article were obtained from the Adolescent Brain Cognitive Development (ABCD) Study (https://abcdstudy.org), held in the NIMH Data Archive (NDA). This is a multisite, longitudinal study designed to recruit more than 10,000 children age 9–10 and follow them over 10 years into early adulthood.
